# Identifying potential indicators to measure the outcome of translational cancer research: a mixed methods approach

**DOI:** 10.1186/s12961-015-0060-5

**Published:** 2015-12-03

**Authors:** Frédérique Thonon, Rym Boulkedid, Maria Teixeira, Serge Gottot, Mahasti Saghatchian, Corinne Alberti

**Affiliations:** European and International Affairs Unit, Gustave Roussy, Villejuif, France; AP-HP, Hôpital Robert Debré, Unité d’épidémiologie clinique, Paris, France; Université Paris Diderot, Sorbonne Paris Cité, UMR-S 1123 and CIC-EC 1426, ECEVE, Paris, France; INSERM, U 1123 and CIC-EC 1426, ECEVE, Paris, France

**Keywords:** Cancer, Delphi technique, Indicators, Qualitative research, Research impact, Translational research

## Abstract

**Background:**

In a context where there is an increasing demand to evaluate the outcome of bio-medical research, our work aims to develop a set of indicators to measure the impact of translational cancer research. The objective of our study was to explore the scope and issues of translational research relevant to evaluation, explore the views of researchers on the evaluation of oncological translational research, and select indicators measuring the outcomes and outputs of translational research in oncology by consensus.

**Methods:**

Semi-structured interviews amongst 23 researchers involved in translational cancer research were conducted and analysed using thematic analysis. A two-round modified Delphi survey of 35 participants with similar characteristics was then performed followed by a physical meeting. Participants rated the feasibility and validity of 60 indicators. The physical meeting was held to discuss the methodology of the new indicators.

**Results:**

The main themes emerging from the interviews included a common definition for translational research but disagreements about the exact scope and limits of this research, the importance of multidisciplinarity and collaboration for the success of translational research, the disadvantages that translational research faces in current evaluation systems, the relative lack of pertinence of existing indicators, and propositions to measure translational cancer research in terms of clinical applications and patient outcomes. A total of 35 participants took part in the first round survey and 12 in the second round. The two-round survey helped us select a set of 18 indicators, including four that seemed to be particularly adapted to measure translational cancer research impact on health service research (number of biomarkers identified, generation of clinical guidelines, citation of research in clinical guidelines, and citation of research in public health guidelines). The feedback from participants helped refine the methodology and definition of indicators not commonly used.

**Conclusion:**

Indicators need to be accepted by stakeholders under evaluation. This study helped the selection and refinement of indicators considered as the most relevant by researchers in translational cancer research. The feasibility and validity of those indicators will be tested in a scientometric study.

**Electronic supplementary material:**

The online version of this article (doi:10.1186/s12961-015-0060-5) contains supplementary material, which is available to authorized users.

## Background

Measuring the long-term impact of translational cancer research, particularly that on health service and patient outcomes, has become of crucial importance. The concept of translational research has been described by Elias Zerhouni, the former director of the National Institute of Health, as “*crossing the valley of death*” between biomedical research and clinical applications [1], and has been formally defined as “*research that transforms scientific discoveries arising in the lab, clinic, or population into new clinical tools and applications that reduce cancer incidence, morbidity, and mortality*” [2]. While this type of research, focused on improving patient outcomes, has increasingly become the centre of attention [3], there has meanwhile been an increase in the demand to measure the outcome of biomedical research in terms of patient benefits and move beyond classical bibliometric indicators [4]. Many professional bodies have developed indicators for various fields, with the goals of improving quality by detecting suboptimal performance based on the traditional Donabedian model, which assesses structures, processes and outcomes [5]. According to Pozen et al. [6], indicators can help track a translational research organisation’s progress towards goals, as well as highlight achievements and identify areas for improvement.

Our work is part of a broad initiative to assess the quality of translational oncological research. Other researchers have focused on developing an assessment framework, called the Excellence Designation System, containing 18 criteria to identify excellent comprehensive cancer centres [7]. We aim to develop metrics to measure the outcome and impact of translational cancer research. In this article, we make a distinction between output and outcome indicators. We define output as “*the immediate tangible result of an activity*” and outcomes as “longer term effects such as impact on health” [8]. Impact is defined as “*the overall results of all the effects of a body of research have on society. Impact includes outputs and outcomes, and may include additional contributions to the health sector or society*” [9].

We first performed a systematic review of indicators measuring the outcome of biomedical research, including their methodology, use, and positive and negative points [10]. We found a total of 57 indicators classified into six categories: indicators of scientific production and impact, indicators of collaboration, indicators of industrial production, indicators of dissemination, and indicators of health service impact. The vast majority of indicators found were bibliometric indicators measuring scientific production and impact. Given the important number of indicators retrieved, it is now necessary to select the ones that are the most relevant to evaluate the outcome of translational cancer research.

The OECD states three criteria to select indicators, namely the importance of what is being measured, its scientific soundness, and its validity [11]. The importance of what is being measured refers mainly to its policy importance. The scientific soundness of an indicator includes its validity (the capacity of an indicator to measure what it is intended to measure), reliability (its capacity to provide stable results when repeated by different people), and explicitness of the scientific base (scientific evidence to support the use of the indicator). The feasibility of an indicator includes characteristics such as existence of prototype (whether the indicator is already in place), the availability of data source, and the cost and burden of measuring that indicator [11].

The aim of our study was to define a set of indicators to measure the output and outcome of oncological translational research. Specifically, we wanted to explore the scope and issues of translational research relevant to evaluation and the views of researchers on the evaluation of oncological translational research, and to select indicators measuring the outcomes and outputs of translational research in oncology by consensus.

## Methods

### Study design

**Figure 1:**
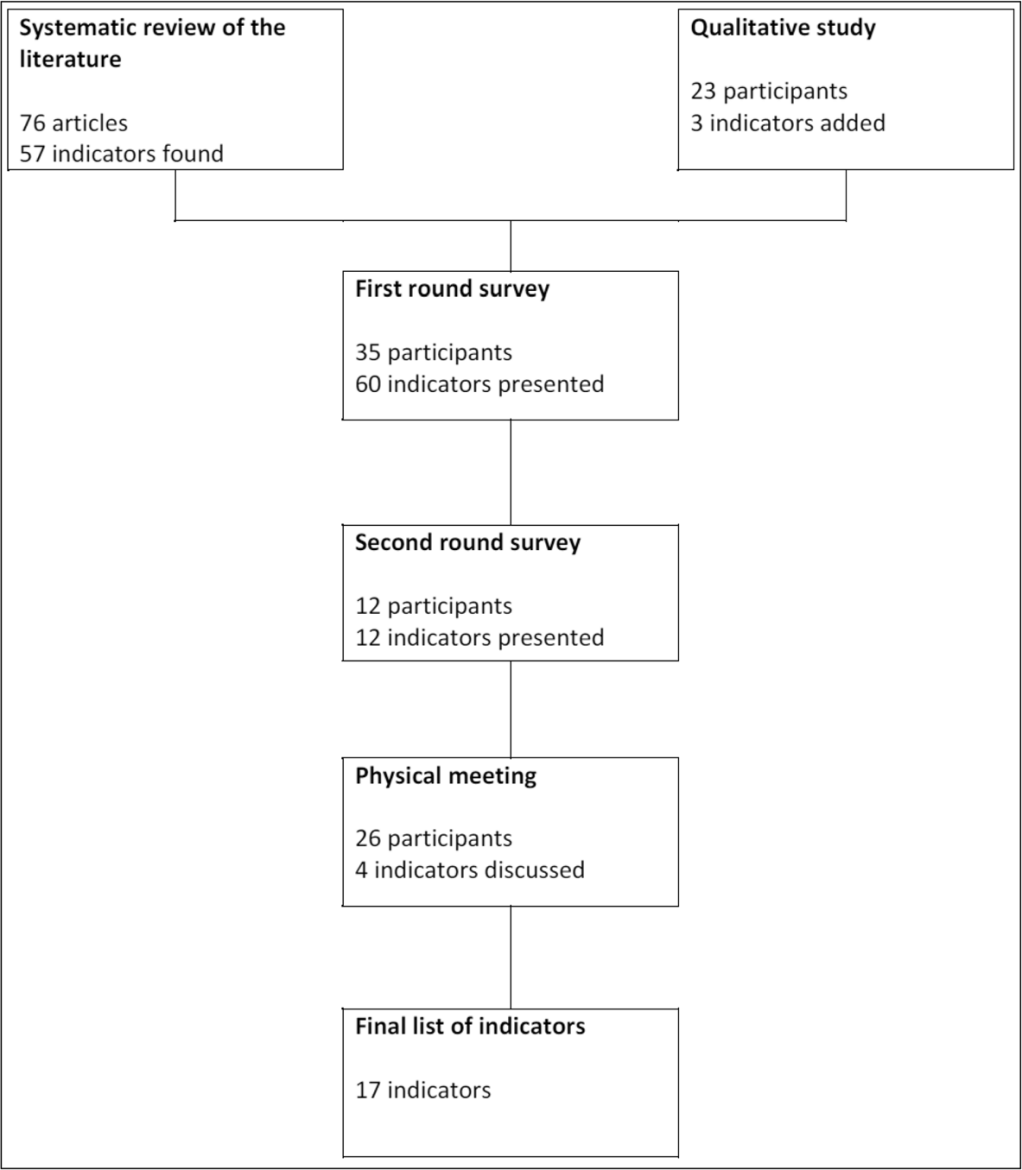
**Indicator development and selection process.**

This study was undertaken as part of a project aiming to develop and test indicators measuring the outcome of translational cancer research. We used several steps to develop and select indicators measuring the outcome of translational research (Figure 1) starting with a systematic literature review whose results are reported in a previous article [10]. We used a mixed-methods approach in order to select a set of indicators to measure the output and outcome of oncological translational research. A mixed-methods design is defined as a design that includes at least one quantitative method (collecting numbers) and one qualitative method (collecting words) [12]. As such, the present study involves semi-structured interviews followed by a modified Delphi survey amongst researchers. The Delphi technique is a structured process that uses a series of questionnaires to gather information. Rounds are held until group consensus is reached. A modified Delphi survey is composed of at least two rounds and a physical meeting [13].

### Participants

For the qualitative study, we invited professionals (clinicians, engineers, researchers, administrative staff) working in translational cancer research. Since translational cancer research involves many different disciplines and professionals, we did not select participants according to their qualification but only according to their involvement in translational cancer research. We defined involvement in translational cancer research as either of the following:

- Working in a research department or unit clearly identified as a conducting translational oncological research (e.g. translational research platform of a cancer hospital)
- Involved in a consortium clearly identified as a translational cancer research consortium (e.g. EurocanPlatform or ERA-NET on translational cancer research)
- Having presented their work in a translational cancer research conference (e.g. the ‘translational research’ session of the European Society for Medical Oncology or American Society of Clinical Oncology conference)
- Teaching in a course or training on translational research in oncology

In addition, we used the ‘snowballing’ method, asking participants to recommend other participants involved in translational cancer research. To form a diverse sample, we sought to include a wide range of professions and practice disciplines and backgrounds in order to ensure that they will represent all potential differences in background, occupational environment, clinical approaches, or practices. We included participants until the saturation point was reached, i.e. until additional interviews produced no new information. In this type of interviews, the saturation point is usually reached after 20 interviews [14].

For the Delphi survey, we invited all participants of the EurocanPlatform network (n = 267). EurocanPlatform is a consortium of 28 cancer organisations (cancer centres and cancer research institutions) in Europe dedicated to translational research on cancer prevention, early detection and treatment. Differences in the recruitment strategies between the qualitative study and the Delphi survey are explained by the different constraints and objectives of each study. For the qualitative study, we favoured participants with a close geographical location in order to allow face-to-face rather than phone interviews so as to obtain more in-depth information. For the Delphi survey, we restricted participation to staff from our consortium in order to receive a more specific feedback.

### Interviews

We conducted semi-structured interviews (with open questions) of professionals involved in translational oncological research around three key themes: (1) definition and scope of translational research, (2) issues and objectives of translational research, and (3) evaluation of the outcome of translational research. The interview guide was designed by FT, MT and SG and tested amongst four participants meeting the inclusion criteria mentioned above. The study objective was briefly presented to the participants at the beginning of the interviews. Two of those pilot interviews were kept in the final set. As two participants were interviewed conjointly, we conducted 22 interviews face-to-face (n = 18) or by telephone (n = 4) and audio-recorded them. Interviews lasted between 15 and 52 minutes, with an average of 25 minutes. All interviews were fully transcripted verbatim. We used a thematic analysis for our study with the help of NVivo10 software. According to a method proposed by Burnard to increase the validity of findings [15], FT, RB, MT and SG studied a sample of five interview transcripts to identify emerging themes, and then the recurring ones. Those themes were then applied as ‘codes’ used to code interviews. We report the results of this analysis according to the Consolidated Criteria for Reporting Qualitative research (COREQ) guidelines [16] (Additional file 1).

### Survey

We conducted a modified Delphi survey [13] amongst researchers in translational cancer research. We invited 267 members of the project to participate in the survey. Participants were sent the online questionnaire by email, with a reminder email in case of non-response. Participants were presented an initial list of indicators composed of all indicators retrieved from the systematic review of the literature [10] and suggested by researchers in the qualitative study, with the definition and positive and negative points according to the literature. No filter on feasibility or validity was used to pre-select indicators from the initial list. The initial list submitted to participants included a mix of ‘traditional’ indicators measuring research dissemination and other indicators measuring alternative ways to disseminate research results, such as ‘citation of research in clinical guidelines’, ‘citation of research in public health guidelines’, ‘reporting of research in the news/media’ and ‘citation in medical education books’. The questionnaire was previously tested amongst two researchers. Each participant was invited to rate the indicators based on validity and feasibility on a scale of 1–9 (1 for indicators definitely not feasible or valid and 9 for indicators definitely feasible or valid). Each participant was also invited to comment on each indicators using a dedicated ‘comment box’, and/or to suggest indicators considered as important. Participants’ characteristics were noted, including their workplace, number of years of experience and training. The entire questionnaire can be found in Additional file 2.

The definition of a ‘consensus’ among participants in a Delphi survey is not agreed upon, and various definitions have been previously used. We chose to apply the definition used by Shield et al. [17]. The median was used to measure the central tendency for the ratings. The final disposition of each indicator was based on the median validity rating, median feasibility rating, and agreement among panellists expressed as a percentage. A higher agreement rate was required in the second than in the first round, as second-round participants were aware of the survey contents and received feedback about the first round, including the median ratings with the ranges, the participants’ responses, and a summary of all the comments received. These data allowed each participant to assess their position relative to the rest of the group, which may have influenced the response to the second round.

In the first round, we selected indicators for which a consensus was achieved regarding validity and feasibility, i.e. for which the median score was in the top tertile (7–9) and at least 65% of panel ratings were in the top tertile. To be included in the final set, indicators had to have median validity and feasibility ratings in the top tertile (7–9) and 75% agreement among panellists that the rating was in the top tertile. We excluded all other indicators, except those that received significant questions or comments suggesting a change in definition or methodology, which we submitted to the second round with the proposed modifications or clarifications.

On the second round, the questionnaire was personalised for each respondent. Participants received an individualised survey form by email presenting their rating, as well as the average rating and comments for each indicator. They were given the opportunity to re-rate in light of those new elements. For the second round, we asked participants to rate only the validity of the indicators (and not the feasibility) in order to make the questionnaire less burdensome and allow more in-depth feedback on indicators. Each round was carried out over a 2-month period (February–March and May–June 2014).

To refine the indicator development and selection process, we held a physical meeting of participants to discuss indicators for which there was debate on the methodology or calculation.

### Ethics statement

The qualitative study and the Delphi survey did not involve patients and written consent was not required. We ensured confidentiality for all participants. We described the study objective and confidentiality treatment before the start of each interview and at the beginning of the survey, and subsequently requested the participant’s consent for audio recording. We anonymised all data linked to participants in the final data set.

## Results

### Characteristics of participants

#### Participants in the qualitative study

We invited 31 actors of translational research in oncology to take part in our study, eight of whom refused due to time constraints or did not reply. A total of 23 researchers, engineers, clinicians or administrators were finally included, amongst them seven women and 16 men. We reached the saturation point after 23 participants. The number of years they worked in cancer research ranged from 5 to 37 years, with a median of 19 years. We interviewed a mix of medical doctors (n = 10), pharmacists (n = 6) and scientists (n = 7), holding degrees in disciplines as diverse as physics, biology and chemistry. The characteristics of participants are shown in Table 1.

**Table 1:** **Characteristics of participants in each survey**

	**Qualitative study n (%) (n = 23)**	**Delphi first round n (%) (n = 35)**
Years of experience in cancer research		
5–9	7 (30)	5 (14)
10–14	2 (9)	4 (12)
15–19	5 (22)	4 (12)
20–24	2 (9)	11 (31)
25–29	5 (21)	6 (17)
≥30	2 (9)	5 (14)
Sex		
Male	16 (70)	24 (69)
Female	7 (30)	11 (31)
Institution of affiliation type		
Comprehensive cancer centre	11 (48)	17 (49)
University hospital	6 (26)	11 (31)
Public research institute (not affiliated to a hospital or cancer centre)	2 (9)	4 (11)
National agency	2 (9)	0
International or European agency	2 (9)	0
Industry (pharmaceutical or consulting)	0	3 (9)
Country		
French institution	18 (79)	3 (9)
Italian institution	1 (4)	8 (23)
Dutch institution	0	6 (17)
British institution	0	4 (11)
Belgian institution	0	3 (9)
Other European country institution	3 (13)	11 (31)
International institution	1 (4)	0
Training		
Masters degree (MSc)	0	1 (3)
Medical degree (MD)	2 (9)	7 (20)
Medical degree and MSc	1 (4)	0
Medical degree and PhD	7 (30)	14 (40)
Pharmacy degree (PharmaD)	1 (4)	0
Pharmacy degree and PhD	5 (22)	1 (3)
PhD	7 (30)	12 (34)
PhD or MSc specialisation	20	28
Biology	10 (50)	13 (46)
Chemistry or biochemistry	2 (10)	2 (7)
Pharmacology	2 (10)	0
Physics	2 (10)	2 (7)
Computer sciences	1 (5)	0
Management of healthcare organisations	0	2 (7)
Immunology	1 (5)	2 (7)
Genetics	1 (5)	3 (10)
Epidemiology/statistics	0	2 (7)
Biotechnology	1 (5)	1 (4)
Pathology	0	1 (4)

Note: Two participants took part in both studies.

#### Participants in the modified Delphi survey

Of the 267 participants invited to complete the survey, 35 participated in the first round, of whom 69% were men (n = 24) and 31% women (n = 11). Most of the participants had a degree either in medicine (n = 21) or/and biology (n = 13). The number of years they worked in cancer research ranged from 4 to 40 years, with a median of 25 years. The participants were affiliated to 21 different institutions out of the 28 of the consortium. The characteristics of participants are described in Table 1.

Two researchers participated in both the qualitative study and Delphi survey (one clinician-researcher and one biologist). One pharmacist participated in the pilot of the qualitative study and the Delphi survey.

### Themes and subthemes extracted from the qualitative study

We extracted themes and sub-themes from the interviews. The main themes from the interviews were (1) definition of translational research; (2) the organization of translational cancer research; (3) the importance of multidisciplinarity and collaboration for the success of translational research; (4) the optimal conditions and barriers for conducting translational research; and (5) issues related to the evaluation of translational research.

Herein, we present the themes most relevant to our study objectives and the selection of indicators, namely definition of translational research, necessity of multidisciplinarity and collaboration, and issues related to the evaluation of translational research. We summarised and grouped the themes ‘definition’ and ‘multidisciplinarity’ under the same sub-part ‘issues to consider when developing an evaluation system’ and presented the ‘issues related to evaluation’ as a single sub-part because we thought this last theme was of particular significance and needed to be more detailed.

### Issues to consider when developing an evaluation system (qualitative study)

#### Definition of translational research: a common basis but unclear limits and scope

The majority of participants of the qualitative study subscribed to a ‘bench-to-bedside’ definition of translational research, meaning a translation of basic findings into clinical research. However, some discrepancies could be observed in terms of what exactly was included in the definition of translational research. Discrepancies were found in the following areas: whether or not translational research is a two-way process (including bedside-to-bench process) or a one-way process (restricted to bench-to-bedside); whether translational research stops at the translation to clinical research or if it includes translation of research findings to clinical practice, whether translational research includes pre-clinical research on mouse models or whether it is restricted to research on human subjects or samples, and whether or not research in epidemiology or cancer prevention is part of translational research.

The majority of participants described translational research as the discipline transferring findings from basic or fundamental science into clinical science without spontaneously mentioning the second aim of translational research, which is to use clinical observation to enrich fundamental research. Five researchers explicitly stated the bi-directional characteristic of translational research on the basis that the objectives of translational research are both to better treat cancer patients and to develop our understanding of cancer. Two researchers rejected this bi-directional definition of translational research. One of them mentioned:

“*I tend to consider translational research in one way only. From the lab to the clinic. It is true that there might be some disciplines where the other way, from the clinic to the lab, can also be done. But as I tend to consider it one way only, I think we shouldn’t keep on funding laboratories of so-called translational research that don’t translate anything.*” (Clinician-researcher, university hospital, 21 years of experience in cancer research)

While most participants mentioned translational research as the translation from basic research to clinical research, only two participants also mentioned the translation of research into clinical practice.

There was also disagreement about whether or not translational research was restricted to research carried out in humans or if it included pre-clinical research such as mouse models. While some participants explicitly stated that translational research was restricted to the research carried out in humans or human samples, others identified their research as translational research, even if it was not carried out on human samples or on patients. Finally, while most participants defined translational research as having therapeutic aims, one participant insisted on the inclusion of research into risk factors and epidemiology as part of translational research.

It should also be noted that two participants rejected the notion of translational research, as an ill-defined concept. As one researcher mentioned:

“*People put whatever they want* [in the definition of translational research] *because it does not really mean anything. It is a trendy word because every other year we need a new fancy word. It used to be ‘transfer research’ then ‘applied research’.*” (Clinician-researcher, university hospital, 37 years of experience in cancer research)

Some researchers (n = 4) expressed concerns about a lack of interest for some disciplines within the translational research continuum, namely imaging, radiotherapy and epidemiology/prevention. One participant mentioned the influence of industry and the donors’ desire to find a cure for cancer as reasons for the imbalance between research in epidemiology/prevention and therapeutic research:

“*There is much more investment into clinical research than in epidemiological and prevention research. There are two reasons for that: the first is that the public wants a cure to cancer.* […] *Charities funding cancer research adapt their priorities to the requests of donors. The second reason is financial. Epidemiological research does not profit companies, although it saves the government money. There should be a stronger public support for epidemiological research, as clinical research is already supported by industries. Charities and the public should be more biased towards epidemiological research.*” (Researcher, public research institute, 26 years of experience in cancer research)

#### Multidisciplinarity and collaboration are crucial for the successful conduct of translational research

All participants mentioned the importance of collaboration as an essential condition for translational research. It was usually considered as the most important issue in the conduct of translational research. In this context, collaboration meant collaboration between clinicians and fundamental researchers, integration of professionals from various medical disciplines in a translational research project (such as pathologists, radiologists, etc.) or, to a lesser extent, collaboration between different institutions, particularly concerning rare or childhood cancers. The collaboration between clinicians and fundamental researchers was the most often cited. Participants who mentioned this type of collaboration argued that clinicians tend to be more aware about patients’ issues and hospital constraints, while basic researchers were said to be more realistic regarding the issue of a project. One participant summarised:

“*Sometimes people who do fundamental research, it is good, they have ideas, but eventually, they have no idea about what is a patient, or the same difficulties* [we encounter as clinicians]. *And I think that the clinician’s perspective can make research more practical, more applied, closer to the issues. On the contrary, those who carry out fundamental research, because they don’t have this commitment towards the patient, are maybe more reasonable regarding what is possible and not possible, more practical in the delays of application* […]. *Or on the contrary more unreasonable and follow implausible paths that sometimes lead to nothing, sometimes there are good ideas that clinicians have not thought of as they keep their nose to the grindstone.*” (Clinician-research, university hospital, 16 years of experience in cancer research)

### Views of researchers on evaluation of translational cancer research and indicators (qualitative study)

#### Existing evaluation systems reward translational research less favourably

Several researchers (n = 5) felt that the indicators or evaluation systems currently in place rewarded translational research less favourably compared with other types of research. Participants perceived that basic or fundamental research was seen as more ‘intellectual’, and therefore of higher value.

A commonly cited example of this bias was the publication of the results of translational research in prestigious or high-impact factor journals. The impact factor is an indicator of journal visibility that is based on the ratio of the number of citations to the number of citeable items of a journal. Participants mentioned that results of translational research tend to be published in journals with a lower impact factor than results of fundamental/basic research. One participant argued that there is not yet a real scientific support for good or very good translational studies. Another researcher argued that multidisciplinarity is less well evaluated than mono-thematic research.

As a consequence, physicians and researchers had fewer incentives to develop translational research projects, as they were seen as ‘less profitable’ or even ‘risky’ for their career, according to participants. One researcher explained that

“*We expect translational research to have much more impact on treatments and patients care, all medical aspects that fundamental research… OK there can be a researcher that spends a lot of time, even all his life, researching on things that will never be applied. Translational research is applied research. Which means there are specific evaluation criterions to this applied research. In particular the impact in terms of health that this translational research should have.*” (Clinician-researcher, comprehensive cancer centre, 25 years of experience in cancer research)

#### Classical indicators are acceptable but not sufficient

Opinions were divided about the utility of existing indicators and evaluation systems, with some participants positive to those systems and others opposed. The existing systems we refer to are those routinely used to assess the performance of researchers and mainly based on bibliometric indicators such as the number of publications or impact factor. The most commonly stated opinions on those indicators can be summarised as, “*existing indicators are acceptable but not sufficient to measure biomedical research/translational research*” and “*existing indicators are not adapted to measure biomedical translational research*”.

The impact factor was the indicator that attracted most critics from participants. One participant felt that it was detrimental to some disciplines where scientific articles are not generally published in high impact factor journals. Two participants noted that negative results are less likely to be published in high impact factor journals:

“*Sometimes a translational research project is a project that does not confirm a data.* […] *We talked a lot about MET for head and neck cancer, we wanted to explore this marker and see if we could offer treatment against this oncogene.* […] *We came to the conclusion that there was no MET abnormality and it was not interesting to develop clinical strategies for that. We had a lot of trouble publishing those data because they were negative.*” (Engineer, comprehensive cancer centre, 16 years of experience in cancer research)

Other participants (n = 5) mentioned research discoveries that either had a significant impact on patient care but were not published in prestigious journals or, on the contrary, had very favourable bibliometric indicators but a very limited impact on patient health or that never even had any clinical application. One participant argued that publishing in high impact factor journals was a waste of time, as it required adding an important number of pointless data. Therefore, publishing in low impact factor journals was a chosen strategy and, thus, the impact factor an inadequate measure of the quality of research:

“*The only difference between a journal with a high impact factor and a journal with an average impact factor is the quantity of data that you add to strengthen you hypothesis. But if your hypothesis is strong with a small number of figures, you don’t need to spend two additional years on the same hypothesis.*” (Clinician-researcher, comprehensive cancer centre, 7 years of experience in cancer research)

Participants did not mention whether publishing in open access journals made any difference. Patent count was also criticised. According to two researchers, a patent only measured a discovery, but a discovery may have no impact or never be translated into applications. A participant argued that patent count could hinder collaboration between institutions and researchers, and another that patentability is less important in translational research than in basic research.

“*This indicator* [patent count] *is pretty good because it measures innovation and clinical applications. But it has negative consequences because it can discourage collaboration. It can incite researchers to keep their biological material and not share it.*” (Pharmacologist, research agency, 26 years of experience in cancer research)

Finally, publication count was mentioned as pushing researchers to move on to another subject rather than ensuring translation of results into applications and citation count was seen as an indicator detrimental to some disciplines.

#### Measuring translational research in terms of clinical applications or patient outcomes

Many participants (n = 15) stated that translational research should ideally be evaluated in terms of applications produced, changes in clinical practice or patient benefits.

The applications mentioned by participants included clinical studies generated as a result of translational findings or to validate those findings, bioinformatics tools created, valid biomarkers developed, databases generated, patents, translation of research findings into treatment protocols or changes in clinical practice, or reduction in waiting time to obtain results. One researcher suggested using indicators specific to translational research protocols (such as the number of patients included) as means to evaluate global translational activity. However, one clinician-researcher warned against the sole count of biomarkers as some developed biomarkers may prove invalid and their development could lead to a loss of opportunity for patients participating in subsequent clinical trials testing this biomarker.

“*Let’s take the example of ERCC1, which produced two ‘New England Journal of Medicine’, one therapeutic trial and eventually had not clinical application. In your criteria, it comes off at the top of the list, there is a patent on it… it ticks all the boxes. But there was no clinical application; it is something that was dropped.* […] *In practice not only it has not been developed, but on top of that there has been potentially a loss of chance since we used a biomarker that was retrospectively invalidated.*” (Clinician, comprehensive cancer centre, 9 years of experience in cancer research)

Most participants argued that translational research should ideally be measured in terms of patient benefits. The exact definition of what constituted patient benefits was not specified and, when specified, could differ between participants. Survival of the patient, but also shortening the treatment duration, diminution of doses, and less side-effects were mentioned as possible clinical results to be included. One clinician involved in paediatric research mentioned that, for some tumours, the survival rate is 90%. However, the issue of long-term toxicities in adult life was an issue of growing importance. Finally, one participant stated the diminution of treatment costs for healthcare systems as another proxy on which to evaluate the success of translational research.

We compiled all suggestions of measure by participants and the corresponding indicators that could be used in Table 2. We added those indicators to the Delphi survey if the definition was clear enough.

**Table 2:** **Suggestions of indicators by participants of the qualitative study**

**Suggestion or comment by participant**	**Possible indicator (according to author)**	**Authors’ comments**
“*Collaboration between biologists and epidemiologists is important and should be measured in terms of outputs, such as joint papers*”	Number of publications co-authored by an epidemiologist and a biologist	This is the only indicator of multidisciplinarity proposed by a participant; however, it is very specific to research in molecular epidemiology
		No similar indicator has been created; indicator added to the Delphi survey
“*One interesting indicator would be the number of patients in a clinical trial benefiting from a biomarker identification*”	Number of patients included in a clinical trial with a biomarker identification	That indicator would be studied by a survey of cancer centres; indicator added to the Delphi survey
“*The point of translational research is to transfer to clinical practice. So it is supposed to generate clinical studies. Ideally it* [an evaluation measure] *would be how many positive studies had been generated*”	Number of hypotheses generated	Literature suggests one indicator of ‘number of hypotheses generated’ [6], but no methodology is proposed; indicator added to the Delphi survey
“*A good indicator of translational research would be its capacity to generate hypothesis to test in the clinic.* […] *So the protocols of clinical validation that have been generated*”		
“*We should ensure whether the tools developed are effective enough to process data the correct way*”	Measures of effectiveness of developed tools	The participant clearly specified that it was an indicator specific to their field (bioinformatics) and not applicable to whole translational research; not added to the questionnaire due to lack of clear definition
“*The primary aim* [of translational research] *would be to adapt technologies to the general population. So it should be evaluated on this aspect*”	Use of developed technologies in practice	No existing indicator; not added to the questionnaire due to lack of clear definition
“ *Developing a biomarker in translational research will help to select patients that will benefit from a treatment, that is a real proxy of translational research efficacy*”	Number of biomarkers developed	Literature suggests one indicator of ‘number of biomarkers identified’ [6], but no methodology is proposed; indicator already part of the Delphi survey
“*What should be measured, for translational research, is the benefit for the patient. Not the final benefit* […] *but the interim benefit, such as biomarkers developed*”		
“*The ideal for translational research, it that it modifies patient care. So that can be a long-term objective, but* […] *if there are interim step*”		
“*Ideally, a translational study should lead to an application, which means, from clinical to basic research, to a fundamental research project, and in the opposite direction, to a clinical application, such as a clinical trial, the validation of a biomarker, or an imaging study*”		
“[translational research should be evaluated] *in terms of publications and implementations in the clinics.* […] *Also guidelines*”	Clinical guidelines generated	There are two existing indicators measuring the transfer of research in clinical guidelines: number of clinical guidelines generated and citation of research in clinical guidelines; indicators already part of the Delphi survey
“*The development of database is also an important structural factor… an indicator*”	Number of databases created	Literature suggests one indicator of ‘number of databases created’ [6], but no methodology is proposed; indicator added to the Delphi survey

### Selection of indicators (survey)

#### First round survey

In the first round, we presented 60 indicators to a panel of 35 participants. Table 3 shows the rating of each indicator by participants. Forty-two indicators were excluded, 12 were submitted to a second round, and six were included in the final set without being submitted to a second round. The six indicators included at the end of the first round were number of clinical trials, percentage of patients included in a clinical trial, number of peer-reviewed publications, number of citations, number of public-private partnerships, and impact factor. They were included in the final set without being submitted to a second round because they received an unusually high rating for feasibility and validity (≥75%).

**Table 3:** **Rating and selection of indicators by participants to the Delphi survey**

	**Round 1**			**Round 2**	
**Indicator**	**Feasibility**	**Validity**	**Status**	**Validity**	**Status**
Number of clinical trials	81%	84%	Included	–	Included
Percentage of patients included in a clinical trial	77%	81%	Included	–	Included
Number of peer-reviewed publications	95%	81%	Included	–	Included
Number of citations	88%	85%	Included	–	Included
Number of public-private partnerships	75%	78%	Included	–	Included
Impact factor	96%	76%	Included	–	Included
Institutional h-index	92%	73%	Second round	83%	Included
Number of publications co-authored with another organisation	92%	72%	Second round	76%	Included
Mean number of citations per article	91%	69%	Second round	66%	Included
Number of highly cited publications	78%	67%	Second round	69%	Included
Number of publications in top-ranked journals	85%	65%	Second round	69%	Included
z-index	83%	71%	Second round	69%	Included
Number of publications with international collaboration	92%	67%	Second round	85%	Included
Number of biomarkers identified	33%	52%	Modified	58%	To be discussed
Citation of research in clinical guidelines	71%	71%	Second round	62%	To be discussed
Generation of clinical guidelines	74%	67%	Second round	54%	To be discussed
Citation of research in public health guidelines	60%	58%	Modified	50%	To be discussed
Number of patents	88%	65%	Second round	38%	Excluded
Number of patients in a clinical trial with a biomarker identification	56%	58%	Excluded	–	–
Number of biological samples collected	69%	50%	Excluded	–	–
Number of biological samples transmitted	42%	34%	Excluded	–	–
Number of hypotheses generated	46%	46%	Excluded	–	–
Number of diagnostic tests created	50%	45%	Excluded	–	–
Number of database generated	50%	38%	Excluded	–	–
Number of research projects ongoing	57%	29%	Excluded	–	–
Number of assays developed	0%	0%	Excluded	–	–
Number of visits to EXPASY server	75%	0%	Excluded	–	–
Clinicians’ awareness of research results	22%	36%	Excluded	–	–
Changes in clinical practices	21%	43%	Excluded	–	–
Contribution to reports informing policy makers	58%	35%	Excluded	–	–
Number of presentations at key selected conferences	60%	56%	Excluded	–	–
Citation in medical education books	42%	48%	Excluded	–	–
Number of conferences held	71%	38%	Excluded	–	–
Citation of research in the media	41%	28%	Excluded	–	–
Number of papers co-authored with the industry	74%	61%	Excluded	–	
Citation of research in patents	79%	58%	Excluded	–	–
Patent h-index	52%	52%	Excluded	–	–
Number of patent citations	73%	48%	Excluded	–	–
Number of spin-off companies created	86%	43%	Excluded	–	–
Partnership-ability index	82%	50%	Excluded	–	–
Dependence degree (d-index)	67%	50%	Excluded	–	–
Proportion of long-distance collaborative publications	43%	0%	Excluded	–	–
Number of publications co-authored by an epidemiologist and biologist	52%	41%	Excluded	–	–
Age-weighted citation rate	71%	33%	Excluded	–	–
b-index	83%	43%	Excluded	–	–
Central index	60%	50%	Excluded	–	–
CH-index	50%	0%	Excluded	–	–
Crown indicator	33%	0%	Excluded	–	–
e-index	80%	50%	Excluded	–	–
g-index	100%	0%	Excluded	–	–
Hg-index	33%	0%	Excluded	–	–
j-index	75%	33%	Excluded	–	–
m-index	50%	25%	Excluded	–	–
m-quotient	67%	60%	Excluded	–	–
Mean normalised citation score	50%	33%	Excluded	–	–
Q^2^ index	25%	0%	Excluded	–	–
r-index	83%	33%	Excluded	–	–
SP-index	100%	50%	Excluded	–	–
w-index	71%	33%	Excluded	–	–
x-index	50%	0%	Excluded	–	–

Percentage of participants rating the indicator in the top tertile (7–9/9). Included: the indicator is included in the final set. Excluded: the indicator is excluded from the selection. Second round: the indicator is submitted to the second round of rating. Modified: The indicator is submitted to the second round of rating with significant changes in the definition and/or methodology following the respondents’ comments. To be discussed: the methodology of the indicator will be discussed in the expert meeting.

Most indicators (n = 51) received comments from participants. Those comments were used to modify the definition or methodology of those indicators. Nine indicators received no comments. As those indicators were the ones that received low ratings for feasibility and validity, they were discarded without further action. A selection of the most common or most striking comments is presented in Additional file 3. The indicator that received comments from most participants was ‘number of biomarkers identified’ (commented by 16 participants). Following those comments, we modified its definition into ‘number of valid biomarkers identified by the institution and published in a peer-review journal’. Another indicator that received many comments was the ‘number of citations in public health guidelines’ for which many participants asked for clarifications about the methodology and definition.

#### Second round survey

In the second round, we presented 12 indicators for rating. Seven of them were included, one was excluded and four were presented for discussion. The seven indicators included were h-index (for institution), number of publications co-authored with another institution, mean number of citations per article, number of publications in top-ranked journals, z-index (an indicator combining the number of publications and the impact factor of those publications), and number of publications with international collaboration.

#### Physical meeting

During a consortium meeting, we organised a session dedicated to indicators. This session was attended by 26 experts, 14 of whom had participated in the survey and the remaining 12 were representatives of participating institutions. The discussion was focused on four indicators: number of valid biomarkers identified by the institution and published in a peer-review journal, number of citations in clinical guidelines, number of citation in public health guidelines, and number of clinical guidelines authored. The discussion was focused on those four indicators since the methodology and definition of these received the most comments and questions in both rounds of the survey. This meeting was moderated by FT and MS. One of the most important points of discussion concerned the inclusion of clinical guidelines for the indicator ‘number of citations in clinical guidelines’. As a conclusion to this discussion, the expert panel agreed on the necessity to define the objective criterion for the inclusion of clinical guidelines based on their quality. At the end of this meeting, we included the four indicators in the set, with the suggestions of participants. In total, 17 indicators were included in the final set. The final list of selected indicators is detailed in Table 4.

**Table 4:** **Selected indicators**

**Indicator**	**Definition**	**Type of indicator**	**Why it measures the impact of translational research**
Number of clinical trial	Number of clinical trials active in a cancer centre in a year	Process	Those indicators measures how research and care are integrated into a hospital
Percentage of patients included in a clinical trial	Ratio of the number of patients included in a clinical trial to the total number of patients	Process	
Number of peer-reviewed publications	Number of peer-reviewed publications authored by the institution	Output	Peer-reviewed publications play a fundamental role in the circulation and exploitation of knowledge
Number of citations	Number of citations of the published articles of an institution received within a time span	Output	It measures how an article has influenced other scientists and its impact on the advancement of knowledge
Impact factor	The ratio of the number of citations to the number of citable items of a journal	Output	This indicator measures the visibility of the production of a research institute
Institutional h-index	Indicator that combines the number of articles produced by research units and their number of citations	Output	As this indicator combines metrics of quantity and visibility, it measures the possible influence of the entire production of a research institute
Number of public-private partnerships	Number of partnerships between an academic research centre and the industry	Process	Public-private partnerships facilitate the translation of research finding into clinical applications
Mean number of citations per article	A ratio of the number of citations received by an institution to their number of publications	Output	This indicator allows a comparison of the potential influence of an institution adjusted for their age
Number of highly cited publications	Number of published articles with a citation count above a certain threshold	Output	This indicator potentially measures the number of articles that had a high impact in the research community
Number of publications in top-ranked journals	Number of publications in journals with the highest impact factor in the discipline	Output	This indicator allows adjustment for differences in citation practices between disciplines
z-index	An indicator combining the number of publications of an institution and the impact factor of those publications	Output	As this indicator combines metrics of quantity and visibility, it measures the possible influence of the entire production of a research institute
Number of publications co-authored with another organisation	Number of publications co-authored by researchers affiliated to another research institution	Output	Cooperation benefits research by bringing new ideas and methods and helping to reach comprehensive expertise. In cancer research, collaboration between institutions is particularly crucial for research on rare cancers where it can be challenging to include enough patients. Those indicators measure the proportion of research performed during a collaboration
Number of publications with international collaboration	Number of publications co-authored by researchers affiliated to a research institution in another country	Output	
Number of biomarkers identified	Number of valid biomarkers identified by the research institution and published in a peer-review journal.	Outcome	Biomarkers play a fundamental role in developing personalised treatments and possibly improving patient outcomes
Generation of clinical guidelines	Number of clinical guidelines authored by the institution	Outcome	Clinical guidelines facilitate the adoption of research findings into practices and aim to improve care quality Those indicators measure the possible influence of research on the improvement of clinical practices
Citation of research in clinical guidelines	Number of articles cited in clinical guidelines	Outcome	
Citation of research in public health guidelines	Number of articles cited in public health guidelines	Outcome	This indicator measures the possible influence of research findings on public policies (e.g. cancer screening)

## Discussion

### Main findings

The aim of the present study was to explore the definitions and issues of translational cancer research according to its researchers, to explore their views on the evaluation of translational cancer research, and to select indicators measuring the output and outcome of translational cancer research. We found many divergences in the exact scope and limits of translational research despite a common definition. Almost all researchers indicated the necessity of multidisciplinarity and collaboration, particularly between physicians and basic researchers, for the success of translational cancer research. Participants had various opinions about the indicators and evaluation systems currently in place; however, most called for the introduction of indicators and an evaluation system more focused on benefits to patients and applications produced.

Most participants defined translational research as a process of translation of basic findings into clinical applications, but very few included the process of translating research into clinical practice. The first process (from basic science to clinical research) has been defined as ‘T1’, while the second has been defined as ‘T2’ [17]. This study confirms that the T2 definition of translational research is overlooked in favour of the T1 definition. Woolf [18] argues that, as most new drugs only marginally improve efficacy, T2 research has a bigger potential for health and the lack of interest in this part of translational research is problematic for public health.

Although the participants of the qualitative study expressed a wish for the introduction of new indicators measuring the longer-term impact of translational research on patients, the results of the Delphi survey differ, with ‘traditional’ indicators (such as number of publications, number of citations and h-index) having the highest ratings and indicators of health service impact (such as citation in clinical guidelines or number of biomarkers identified) having lower ratings. This is possibly due to the fact that those indicators are not routinely used in the evaluation systems currently in place and hence the less clear methodology and lack of familiarity of researchers with these; this might be linked to the Delphi process itself where the necessity for consensus tends not to favour innovative ideas.

There was a significant focus on biomarkers in both studies. On the one hand, many participants of the qualitative study mentioned the identification of biomarkers as a proxy for the success of translational cancer research; on the other hand, the proposed indicator ‘number of biomarkers identified’ received the most comments during the Delphi survey. Biomarkers are defined by the National Cancer Institute as “*a biological molecule found in blood, other fluids, or tissues that is a sign of a normal or abnormal process or of a condition or disease*” [19]. Biomarkers can have many uses such as estimating the risk of a disease, screening for primary cancers, distinguishing a benign from a malignant tumour, determining prognosis and prediction for patients who have been diagnosed with a cancer, and monitoring disease status [18]. Biomarkers play a significant role in developing personalised treatments [20,21] and possibly improving patient outcomes [22]. Therefore, measuring the development of valid biomarkers seems an interesting indicator to measure the impact of translational cancer research. Although suggested once in the literature [6], this indicator has not been tested or used.

### Strengths and limitations of the study (internal validity)

The present study used a mix of qualitative and quantitative methods to investigate the views of researchers on the evaluation of translational cancer research outcomes. We believe that both methods are complementary and their combined use can enhance results [23]. A further strength is the inclusion of participants from a broad range of specialties and backgrounds in both the qualitative study and the Delphi survey. Participants in both studies were involved in various areas of translational research, such as pre-clinical research, phase one studies, epidemiological research, drug development, and administration/management, and have diplomas in disciplines as diverse as biology, information science, physics, chemistry, pharmacology or medicine. They also represented most of the institutions in the research consortium. It has been argued that having a heterogeneous sample of participants enhances generalisation in a qualitative study [24] as well as the validity of a Delphi survey [13].

Our study also has limitations. There was a low participation rate, both in the first round and second round of the Delphi survey. Indeed, keeping adhesion of panel members is one issue of the Delphi procedure [13]. However, the heterogeneity of the sample, both in terms of background, occupation and institutions of affiliation, reduced the effect of this bias, as explained earlier. Out of the 267 researchers invited for the Delphi survey, only 35 participated in the first round. This figure can be explained by the length of the questionnaire and by the fact there was no pre-selection of participants based on their willingness to be included in the survey. Although it is a low take-up rate, a panel of 35 participants is sufficient for a Delphi survey [13]. The participation rate dropped between the first and second Delphi round. The Delphi can be a long process, participants must complete the questionnaire despite their busy schedules and non-respondents must be contacted. However, despite reminders, some participants did not respond. Although some of the panellists failed to participate in all the steps of the Delphi survey (suggesting some degree of weariness with the process), the participation rate was within the range of previous studies, and incomplete participation does not undermine the validity of the results.

Although our study had a focus on developing indicators measuring patient benefits, the final list contains only a relatively small number of these since most of the indicators of health service impact and patient benefits found in the literature either had little or no methodology provided or received a low rating for feasibility or validity. Some of those indicators could be interesting to develop for a case study analysing the impact of the research of a small number of cancer research institutions. However, they are not suited for a large-scale bibliometric study.

### Strengths and limitations of our study in relation to other studies (external validity)

The divergences in the definitions of translational research by the stakeholders involved have been previously highlighted in the literature [25]. However, the necessity for collaboration between basic researchers and clinicians has already been underlined. Lord et al. [26] and de Bono [27] argue that cancer biologists do not have the same understanding of what constitutes a feasible target or a thorough understanding of clinical issues as clinicians, but nevertheless should have a crucial role in the design and analysis of clinical trials. Pober et al. [28] argue that, traditionally, the responsibility for making the connection between laboratory and clinic has fallen on the clinicians-researchers. However, due to difficulties in recruiting and maintaining statutory physicians-researchers with both clinical and basic science training, creating translational research teams with clinicians and laboratory-based investigators is a viable solution.

In the qualitative study, many researchers argued that translational research does not get published in high impact factor journals, and therefore bibliometric indicators do not favour translational cancer research. This opinion seems to be corroborated by a scientometric study [29] which examined trends in publications of translational research. This article found that amongst the first 20 journals in which most translational results are published, only four had an impact factor above 5 and only one an impact factor above 10. However, that article was not specific to cancer research and did not compare translational research publications with basic research publications.

Compared with the study from Rajan et al. [7], our study holds significant differences. Firstly, the Excellence Designation System proposed relies on peer review by experts, which requires organisation and participant input, while the set of indicators that we have developed can be measured using bibliometric data with minimal effort from participants. We believe both approaches are complementary. Indeed, it has been argued that both peer-review and bibliometrics can offer different and complementary methods to evaluate research [30]. Finally, the Excellence Designation System evaluates the quality of translational cancer research with a focus mostly on structure and process, while the set of indicators we propose is focused on research output and outcome.

### Policy implications

This study has enabled us to explore the scope and issues relevant to the evaluation of the outcome of translational research and to select indicators to measure it. However, to be effective, indicators need to be accepted by the stakeholders who will be evaluated. An important added value from our study is that the feedback from the participants in both the qualitative study and the modified Delphi survey has considerably helped us refine the methodology and definition of the indicators selected. However, some problems remain, mainly with the lack of consensus with the exact scope of translational research. The disagreements between researchers regarding the limits and scope of translational research could pose a problem when designing an evaluation system that would be acceptable to most. For example, some indicators suggested by participants, such as the number of fundamental hypothesis generated, might be rejected by those for whom translational research is a one-way process only from the bench to the bedside. Another issue was the many divergences and discussions concerning some indicators that are not routinely used. Exploring the views of researchers on those indicators once they have been tested and used would be very informing.

## Conclusion

This study has enabled us to receive input from actors in translational cancer research on the important issues related to the evaluation of the selected indicators, and to clarify their methodology. We selected a total of 17 indicators, including several new ones measuring the impact of research in terms of health service and patient outcomes. However, some indicators proposed are not routinely used and many researchers are not familiar with these. Testing this set of indicators will provide a more in-depth analysis on their feasibility and validity. A scientometric study of cancer centres using those indicators is warranted.

## Additional files

Additional file 1:
**Consolidated Criteria for Reporting Qualitative research (COREQ) guideline checklist.** (DOC 55 kb) Additional file 2:
**Delphi first round questionnaire.** (PDF 604 kb) Additional file 3:
**Selection of comments from survey participants.** (DOC 36 kb)
