# Supplementary material for: Identifying potential indicators to measure the outcome of translational cancer research: a mixed methods approach
Source: Health Res Policy Syst. 2015 Dec 3;13:72. doi: 10.1186/s12961-015-0060-5 (PMC4669638; doi:10.1186/s12961-015-0060-5)
Supplement: Additional file 1: — Consolidated Criteria for Reporting Qualitative research (COREQ) guideline checklist. (DOC 55 kb) [file 12961_2015_60_MOESM1_ESM.doc]

**Additional file 1: COREQ Guideline checklist**

| **Domain 1 : Research team and reflexivity** | **Personal characteristics** | |
| --- | --- | --- |
|  | 1. Which author(s) conducted the interview? | Interviews conducted by FT |
|  | 1. What were the researcher’s credentials? | FT: PhD candidate in public health; RB: PhD in epidemiology; SG: MD; MT: PhD in anthropology; MS: MD; CA: MD-PhD |
|  | 1. What was their occupation at the time of the study? | FT: PhD candidate; RB, MT, SG: researchers in public health; MS: medical oncologist; CA: professor of epidemiology |
|  | 1. Was the researcher male or female? | 5 females, 1 male |
|  | 1. What experience or training did the researcher have | Experience in conducting qualitative research (MT; SG), experience in conducting Delphi surveys (RB), expertise in public health (all authors), expertise in translational cancer oncology (MS) |
|  | **Relationship with participants** | |
|  | 1. Was a relationship established prior to study commencement | The interviewer did not know the participants before the study. Two participants taught a course in translational research that the interviewer attended. |
|  | 1. What did the participants know about the researcher? | At the start of the study, the aim of the research project, as well as the objectives of the study was presented. |
|  | 1. What characteristics were reported about the interviewer/facilitator? | Interviewer characteristics were not reported to participants |
| **Domain 2: Study design** | **Theoretical framework** | |
|  | 1. What methodological orientation was stated to underpin the study? | We used thematic analysis |
|  | **Participant selection** | |
|  | 1. How were the participants selected? | Participants were recruited for their experience in translational oncological research. We tried to represent a diversity of profiles and backgrounds. |
|  | 1. How were the participants approached? | Originally by email |
|  | 1. How many participants were in the study? | 23 |
|  | 1. How many participants refused to participate or dropped out? Why? | 8 (time constraints or no explanation given) |
|  | **Setting** | |
|  | 1. Where was the data collected? | In the participant’s office (18 interviews) or on the phone (4 interviews) |
|  | 1. Was anyone else present besides the participants and researcher? | No. One interview involved two participants |
|  | 1. What are the important characteristics of the sample? | Diversity of backgrounds and occupation. The characteristics of the sample are described in table 1 |
|  | **Data collection** | |
|  | 1. Were questions, prompts, guides provided by the author? Was it pilot tested? | The interview guide was read by the interviewer to participants. It was tested amongst 4 participants. |
|  | 1. Were repeat interviews carried out? Details | No repeat interviews. |
|  | 1. Did the researcher use audio or visual recording to collect the data? | 19/22 interviews recorded. 3 interviews not recorded because of material dysfunction. |
|  | 1. Were field notes made during and/or after the interview or focus group? | Notes taken during all interviews. |
|  | 1. What was the duration of interviews or focus groups? | From 15-52 minutes. Average: 25 minutes |
|  | 1. Was data saturation discussed? | Data saturation was discussed with authors after 20 interviews. |
|  | 1. Were transcripts returned to participants for comments and/or correction? | Transcripts not returned to participants |
| **Domain 3: Analysis and findings** | **Data analysis** | |
|  | 1. How many data coders coded the data? | Four authors (FT, RB, MT, SG) created the initial coding tree using 5 sample interview transcripts |
|  | 1. Did authors provide a description of the coding tree? | We do not present the coding tree we created |
|  | 1. Were themes identified in advance or derived from the data? | The themes were derived both inductively and deductively |
|  | 1. What software, if applicable, was used to manage the data? | Use of NVivo software. |
|  | 1. Did participants provide feedback on the findings? | No feedback was obtained from participants |
|  | **Reporting** | |
|  | 1. Were participant quotations presented to illustrate the themes/findings? Was each quotation identified? | We present some quotations to illustrate findings. |
|  | 1. Was there consistency between the data presented and the findings | The data presented and the findings are consistent |
|  | 1. Were major themes clearly presented in the findings? | We present the most important themes related to the study objectives in the findings |
|  | 1. Is there a description of diverse cases or discussion of minor themes? | We report and describe diverse cases |
