# Supplementary material for: Identifying potential indicators to measure the outcome of translational cancer research: a mixed methods approach
Source: Health Res Policy Syst. 2015 Dec 3;13:72. doi: 10.1186/s12961-015-0060-5 (PMC4669638; doi:10.1186/s12961-015-0060-5)
Supplement: Additional file 2: — Delphi first round questionnaire. (PDF 604 kb) [file 12961_2015_60_MOESM2_ESM.pdf]

Indicators of research activity, output and outcomes

Description

The objective of the EurocanPlatform network is to build a strong platform for translational research in oncology in order to share the resources and infrastructures of that research and produce better research outcomes.
Within this project, we are developing a set of indicators designed to evaluate the activity and production of the translational research performed in the platform in terms of benefits for science and society. A draft list of indicators was drawn from a systematic review and interviews with researchers. In order to make a selection within that list and to set up a list of indicators that are acceptable to all researchers involved, we are conducting a Delphi survey amongst researchers in oncology. Your contribution is highly valuable to us and we thank you in advance to take the time to answer this questionnaire.
The Delphi method is widely used for the task of achieving consensus among a group of experts on a particular topic. Two or more questionnaire rounds are completed to achieve a consensus among panellists selected based on expertise in the relevant field. After each round, the respondents are asked to reconsider their answers based on both their own opinion and the group response to the previous round. Therefore, we need to collect some information such as your name and email address in order to send you the second round questionnaire. However your answers will remain strictly confidential.

Please note that the following indicators are indicators measuring activity, outputs and outcomes (and not inputs) of the research carried out by institutions (cancer centres, hospitals, research units but not countries or individual researchers).

Instructions:
- Indicators are classified into 6 categories: indicators of research activity, indicators of collaboration, indicators of dissemination, indicators of industrial production, indicators of research outcome/impact and bibliometric indicators.
- You will be asked to rate each indicator for its feasibility and validity. The feasibility of an indicator refers to the possibility and burden of measuring an indicator. It includes conditions such as data availability, comparability of data across cancer centres and countries, and burden of data collection. The validity of an indicator means its capacity to measure what it is intended to measure.
- You will be asked to select the most important indicators in each category (maximum of 5 by category)
- You will be invited to provide comments on each indicator
- At the end of the survey you will be invited to provide free comments and provide up to 5 additional indicators measuring research impact (in terms of patient’s outcome)

General information

*1. Your name:

2. Your gender:

☐ M

☐ F

3. Your year of birth:

Indicators of research activity, output and outcomes

***4. Your academic background (up to three answers possible):**

- [ ] Medical
- [ ] Pharmacist
- [ ] Biologist
- [ ] Chemist
- [ ] Physicist
- [ ] Nurse
- [ ] Other (please precise)

***5. Please indicate the number of years you have worked in cancer research:**

6. Please indicate your job title:

7. Please indicate your institution:

Indicators of research activity

Definition: Indicators of research activity are proxies to measure the state and progression of the translational research performed in an institution

The feasibility of an indicator refers to the possibility and burden of measuring it. It includes conditions such as data availability, comparability of data across cancer centres and countries, and burden of data collection. The validity of an indicator means its capacity to measure what it is intended to measure.

Indicator: Number of clinical trials

Definition : Number of clinical trials (open to patient's inclusion) active in a cancer centre in a specific year

8. Do you understand this indicator?

- ◯ Yes
- ◯ No

Indicators of research activity, output and outcomes

9. Feasibility

1(definitely not feasible)	2	3	4	5	6	7	8	9 (definitely feasible)
○	○	○	○	○	○	○	○	○

10. Validity

1 (definitely not valid)	2	3	4	5	6	7	8	9 (definitely valid)
○	○	○	○	○	○	○	○	○

11. Comments

Indicator: % of patients included in clinical trial

Definition : Number of patients that participate in a clinical trial/ Number of patients treated at a hospital in a specific year

12. Do you understand this indicator?

○ Yes

○ No

13. Feasiblity

1 (definitely not feasible)	2	3	4	5	6	7	8	9 (definitely feasible)
○	○	○	○	○	○	○	○	○

14. Validity

1 (definitely not valid)	2	3	4	5	6	7	8	9 (definitely valid)
○	○	○	○	○	○	○	○	○

15. Comments

Indicator: Number of biomarkers identified

Definition : Number of biomarkers identified by the research institution. A biomarker is ‘a biological characteristic that is objectively measured and evaluated as an indicator of normal biological processes, pathogenic processes, or pharmacologic responses to a therapeutic intervention’

Indicators of research activity, output and outcomes

16. Do you understand this indicator?

- Yes
- No

17. Feasibility

1 (definitely not feasible)	2	3	4	5	6	7	8	9 (definitely feasible)

18. Validity

1 (definitely not valid)	2	3	4	5	6	7	8	9 (definitely valid)

19. Comments

Indicator : Number of patients in a clinical trial that include biomarker i...

Definition : Number of patients in a clinical trial that include biomarker identification/ number of patients in a clinical trial in a specific year

20. Do you understand this indicator?

- Yes
- No

21. Feasibility

1 (definitely not feasible)	2	3	4	5	6	7	8	9 (definitely feasible)

22. Validity

1(definitely not valid)	2	3	4	5	6	7	8	9 (definitely valid)

23. Comments

Indicator: Number of biospecimen collected

Definition : Number of biospecimens (materials taken from the human body, such as tissue, blood, plasma, and urine) that can be used for cancer diagnosis and analysis) collected in a cancer institution per year

24. Do you understand this indicator?

- ◯ Yes
- ◯ No

25. Feasibility

1 (definitely not feasible)	2	3	4	5	6	7	8	9 (definitely feasible)
◯	◯	◯	◯	◯	◯	◯	◯	◯

26. Validity

1 (definitely not valid)	2	3	4	5	6	7	8	9 (definitely valid)
◯	◯	◯	◯	◯	◯	◯	◯	◯

27. Comments

Indicator: Number of diagnostic test created

Number of diagnostic tests developed over a period of 3 years.

28. Do you understand this question?

- ◯ Yes
- ◯ No

29. Feasibility

1 (definitely not feasible)	2	3	4	5	6	7	8	9 (definitely feasible)
◯	◯	◯	◯	◯	◯	◯	◯	◯

30. Validity

1 (definitely not valid)	2	3	4	5	6	7	8	9 (definitely valid)
◯	◯	◯	◯	◯	◯	◯	◯	◯

31. Comments

Indicator: Number of hypotheses generated

Definition : Number of research hypotheses translated in approved research proposals over a period of 3 years

Indicators of research activity, output and outcomes

32. Do you understand this indicator?

- ◯ Yes
- ◯ No

33. Feasibility

1 (definitely not feasible)	2	3	4	5	6	7	8	9 (definitely feasible)
◯	◯	◯	◯	◯	◯	◯	◯	◯

34. Validity

1 (definitely not valid)	2	3	4	5	6	7	8	9 (definitely valid)
◯	◯	◯	◯	◯	◯	◯	◯	◯

35. Comments

Indicator: Number of assays developed

Definition : Number of assays developed by an institution over the last 3 years

36. Do you understand this indicator?

- ◯ Yes
- ◯ No

37. Feasibility

1 (definitely not feasible)	2	3	4	5	6	7	8	9 (definitely feasible)
◯	◯	◯	◯	◯	◯	◯	◯	◯

38. Validity

1 (definitely not valid)	2	3	4	5	6	7	8	9 (definitely valid)
◯	◯	◯	◯	◯	◯	◯	◯	◯

39. Comments

Indicator: Number of databases generated

Number of research databases created by an institution over a period of 3 years. By database we mean a repository that aggregate clinical and molecular data.

Indicators of research activity, output and outcomes

40. Do you understand this indicator?

- Yes
- No

41. Feasibility

1 (definitely not feasible)	2	3	4	5	6	7	8	9 (definitely feasible)
○	○	○	○	○	○	○	○	○

42. Validity

1 (definitely not valid)	2	3	4	5	6	7	8	9 (definitely valid)
○	○	○	○	○	○	○	○	○

43. Comments

Indicator: Number of research projects ongoing

44. Do you understand this indicator?

- Yes
- No

45. Feasibility

1 (definitely not feasible)	2	3	4	5	6	7	8	9 (definitely feasible)
○	○	○	○	○	○	○	○	○

46. Validity

1 (definitely not valid)	2	3	4	5	6	7	8	9 (definitely valid)
○	○	○	○	○	○	○	○	○

47. Comments

Indicator: Number of spin-off companies created

Definition : Number of spin-off created originating from an institution over a period of 3 years

Indicators of research activity, output and outcomes

48. Do you understand this indicator?

- Yes
- No

49. Feasibility

1 (definitely not feasible)	2	3	4	5	6	7	8	9 (definitely feasible)

50. Validity

1 (definitely not valid)	2	3	4	5	6	7	8	9 (definitely valid)

51. Comments

Indicator: Number of visits to the EXPASY server

The EXPASY (Expert Protein Analysis System) is a virtual research infrastructure for bioinformatics. This indicator measures the number of visits to this platform in a specific year

52. Do you understand this indicator?

- Yes
- No

53. Feasibility

1 (definitely not feasible)	2	3	4	5	6	7	8	9 (definitely feasible)

54. Validity

1 (definitely not valid)	2	3	4	5	6	7	8	9 (definitely valid)

55. Comments

Indicator selection

Indicators of research activity, output and outcomes

***56. Please select and rank the most important indicators in this category (maximum 5)**

	1 (Most important indicator of the selection)	2	3	4	5 (Least important indicator of the selection)
Number of clinical trials	○	○	○	○	○
% of patients included in clinical trial	○	○	○	○	○
Number of biomarkers identified	○	○	○	○	○
Number of patients in a clinical trial that include biomarker identification	○	○	○	○	○
Number of biospecimen collected	○	○	○	○	○
Number of diagnostic tests developed	○	○	○	○	○
Number of databases generated	○	○	○	○	○
Number of hypotheses generated	○	○	○	○	○
Number of assays developed	○	○	○	○	○
Number of research projects ongoing	○	○	○	○	○
Number of spin-off companies created	○	○	○	○	○
Number of visits to EXPASY server	○	○	○	○	○

Indicators of research impact/outcome

Definition: These indicators measure the final impact of research, or how much it results in changes in patients' outcomes, health care organisation, public health…

The feasibility of an indicator refers to the possibility and burden of measuring it. It includes conditions such as data availability, comparability of data across cancer centres and countries, and burden of data collection. The validity of an indicator means its capacity to measure what it is intended to measure.

Indicator: Citation of research in clinical guidelines

Definition : Number of articles that are cited in clinical guidelines published in the last 5 years

57. Do you understand this indicator?

○ Yes

○ No

Indicators of research activity, output and outcomes

58. Feasibility

1 (definitely not feasible)	2	3	4	5	6	7	8	9 (definitely feasible)

59. Validity

1 (definitely not valid)	2	3	4	5	6	7	8	9 (definitely valid)

60. Comments

Indicator: Citation of research in public health guidelines

Definition : Number of articles that are cited in policy or public health guidelines published in the last 5 years

61. Do you understand this indicator?

- Yes
- No

62. Feasibility

1 (definitely not feasible)	2	3	4	5	6	7	8	9 (definitely feasible)

63. Validity

1 (definitely not valid)	2	3	4	5	6	7	8	9 (definitely valid)

64. Comments

Indicator: Generation of clinical guidelines

Definition : Number of clinical guidelines published in the last 3 years authored by researchers from an institution

65. Do you understand this indicator?

- Yes
- No

Indicators of research activity, output and outcomes

66. Feasibility

1 (definitely not feasible)	2	3	4	5	6	7	8	9 (definitely feasible)
○	○	○	○	○	○	○	○	○

67. Validity

1 (definitely not valid)	2	3	4	5	6	7	8	9 (definitely valid)
○	○	○	○	○	○	○	○	○

68. Comments

Indicator: Changes in clinical practice

Definition : Number of actual changes in clinical practices stimulated by a particular scientific result. We propose to calculate it over a period of 3 years.

69. Do you understand this indicator?

○ Yes

○ No

70. Feasibility

1 (definitely not feasible)	2	3	4	5	6	7	8	9 (definitely feasible)
○	○	○	○	○	○	○	○	○

71. Validity

1 (definitely not valid)	2	3	4	5	6	7	8	9 (definitely valid)
○	○	○	○	○	○	○	○	○

72. Comments

Indicator: Contribution to reports informing policy makers

Definition : Number of policy reports published in the last 3 years written by at least one member of an institution

73. Do you understand this indicator?

○ Yes

○ No

Indicators of research activity, output and outcomes

74. Feasibility

1 (definitely not feasible)	2	3	4	5	6	7	8	9 (definitely feasible)
◯	◯	◯	◯	◯	◯	◯	◯	◯

75. Validity

1 (definitely not valid)	2	3	4	5	6	7	8	9 (definitely valid)
◯	◯	◯	◯	◯	◯	◯	◯	◯

76. Comments

Indicator: Clinicians' awareness of research results

Definition : Number of clinicians having read articles from an institution published in the last 3 years

77. Do you understand this indicator?

◯ Yes

◯ No

78. Feasibility

1 (definitely not feasible)	2	3	4	5	6	7	8	9 (definitely feasible)
◯	◯	◯	◯	◯	◯	◯	◯	◯

79. Validity

1 (definitely not valid)	2	3	4	5	6	7	8	9 (definitely valid)
◯	◯	◯	◯	◯	◯	◯	◯	◯

80. Comments

Indicator selection

Indicators of research activity, output and outcomes

***81. Please select and rank the most important indicators in that category (5 max)**

	1 (Most important indicator)	2	3	4	5 (Least important indicator)
Citation of research in clinical guidelines	◯	◯	◯	◯	◯
Citation of research in public health guidelines	◯	◯	◯	◯	◯
Generation of clinical guidelines	◯	◯	◯	◯	◯
Changes in clinical practice	◯	◯	◯	◯	◯
Contribution to reports informing policy makers	◯	◯	◯	◯	◯
Clinicians' awareness of research results	◯	◯	◯	◯	◯

Indicators of collaboration

Definition: Indicators of collaboration measure the level of collaboration or joint work, either between two or more institutions, or between different research units or scientists of different disciplines within the same unit

The feasibility of an indicator refers to the possibility and burden of measuring it. It includes conditions such as data availability, comparability of data across cancer centres and countries, and burden of data collection. The validity of an indicator means its capacity to measure what it is intended to measure.

Indicator: Partnership Ability Index (PHI-index)

Definition : The PHI index combines the number of co-authors with the frequency of joint activities between him and his co-authors over a period of 3 years

82. Do you understand this indicator?

◯ Yes

◯ No

83. Feasibility

1 (definitely not feasible)	2	3	4	5	6	7	8	9 (definitely feasible)
◯	◯	◯	◯	◯	◯	◯	◯	◯

84. Validity

1 (definitely not valid)	2	3	4	5	6	7	8	9 (definitely valid)
◯	◯	◯	◯	◯	◯	◯	◯	◯

85. Comments

Indicators of research activity, output and outcomes

Indicator: Number of co-authored publication

Definition : Number of articles that have been co-authored with one or more other institutions (such as hospital) over a period of 3 years

*86. Do you understand this indicator?

- Yes
- No

87. Feasibility

1 (definitely not feasible)	2	3	4	5	6	7	8	9 (definitely feasible)

88. Validity

1 (definitely not valid)	2	3	4	5	6	7	8	9 (definitely valid)

89. Comments

Indicator: Number of articles with international collaboration

Definition : The number of publications of an institution that have been co-authored with one or more countries over a period of 3 years

90. Do you understand this indicator?

- Yes
- No

91. Feasibility

1 (definitely not feasible)	2	3	4	5	6	7	8	9 (definitely feasible)

92. Validity

1 (definitely not valid)	2	3	4	5	6	7	8	9 (definitely valid)

93. Comments

Indicators of research activity, output and outcomes

Indicator: Number of articles that involve collaboration between an epidemi...

Definition : Number of publications co-authored by an epidemiologist and a biologist over a period of 3 years

94. Do you understand this indicator?

- Yes
- No

95. Feasibility

1 (definitely not feasible)	2	3	4	5	6	7	8	9 (definitely feasible)

96. Validity

1 (definitely not valid)	2	3	4	5	6	7	8	9 (definitely valid)

97. Comments

Indicator: Proportion of long-distance collaborative publication

Definition : The proportion of the articles published in the last 3 years that have geographical collaboration distance of more than 1000 km

98. Do you understand this indicator?

- Yes
- No

99. Feasibility

1 (definitely not feasible)	2	3	4	5	6	7	8	9 (definitely feasible)

100. Validity

1 (definitely not valid)	2	3	4	5	6	7	8	9 (definitely valid)

101. Comments

Indicator: d-index (dependence degree)

Indicators of research activity, output and outcomes

Definition : Considering a researcher a1 and his co-author a2. the d-index quantifies how much the productivity of all scientific collaboration of a1 differs when a2 is not involved. We propose to calculate it over a period of 3 years

102. Do you understand this indicator?

- Yes
- No

103. Feasibility

1 (definitely not feasible)	2	3	4	5	6	7	8	9 (definitely feasible)
◯	◯	◯	◯	◯	◯	◯	◯	◯

104. Validity

1 (definitely not valid)	2	3	4	5	6	7	8	9 (definitely valid)
◯	◯	◯	◯	◯	◯	◯	◯	◯

105. Comments

Indicator selection

Please select and rank the most important indicators in this category (maximum 5)

*106. Please select and rank the most important indicators in this category

	1 (Most important indicator)	2	3	4 (Least important indicator)
Partnership Ability Index (PHI-index)	◯	◯	◯	◯
Number of co-authored publication	◯	◯	◯	◯
Number of articles with international collaboration	◯	◯	◯	◯
Number of publications that involve collaboration between an epidemiologist and a biologist	◯	◯	◯	◯
d-index	◯	◯	◯	◯

Indicators of dissemination

Definition: Indicators of dissemination measure the way the research results are disseminated or communicated towards the society (or the scientific community)

Indicators of research activity, output and outcomes

The feasibility of an indicator refers to the possibility and burden of measuring it. It includes conditions such as data availability, comparability of data across cancer centres and countries, and burden of data collection. The validity of an indicator means its capacity to measure what it is intended to measure.

Indicator: Citation in medical education books

Definition : Number of articles written by an institution cited in medical education books over a period of 3 years

107. Do you understand this indicator?

- Yes
- No

108. Feasibility

1 (definitely not feasible)	2	3	4	5	6	7	8	9 (definitely feasible)
○	○	○	○	○	○	○	○	○

109. Validity

1 (definitely not valid)	2	3	4	5	6	7	8	9 (definitely valid)
○	○	○	○	○	○	○	○	○

110. Comments

Indicator: Number of presentations at key selected conference

Definition : Number of presentations at key selected conference by the institution over a period of 3 years

111. Do you understand this indicator?

- Yes
- No

112. Feasibility

1 (definitely not feasible)	2	3	4	5	6	7	8	9 (definitely feasible)
○	○	○	○	○	○	○	○	○

113. Validity

1 (definitely not valid)	2	3	4	5	6	7	8	9 (definitely valid)
○	○	○	○	○	○	○	○	○

114. Comments

Indicator: Number of conference held

Definition : Number of conference held by the institution over a period of 3 years

115. Do you understand this indicator?

- Yes
- No

116. Feasibility

1 (definitely not feasible)	2	3	4	5	6	7	8	9 (definitely feasible)
○	○	○	○	○	○	○	○	○

117. Validity

1 (definitely not valid)	2	3	4	5	6	7	8	9 (definitely valid)
○	○	○	○	○	○	○	○	○

118. Comments

Indicator: Reporting of research in the news/media

Definition : Number of articles cited in the mass media over a period of 3 years

119. Do you understand this indicator?

- Yes
- No

120. Feasiblity

1 (definitely not feasible)	2	3	4	5	6	7	8	9 (definitely feasible)
○	○	○	○	○	○	○	○	○

121. Validity

1 (definitely not valid)	2	3	4	5	6	7	8	9 (definitely valid)
○	○	○	○	○	○	○	○	○

Indicators of research activity, output and outcomes

122. Comments

Indicator selection

*123. Please select and rank the most important indicators in that category

	1 (Most important indicator)	2	3	4 (Least important indicator)
Citation in medical education books	○	○	○	○
Number of presentations at key selected conference	○	○	○	○
Number of conference held	○	○	○	○
Reporting of research in the news/media	○	○	○	○

Indicators of industrial production

Definition: Indicators of industrial production measure the outcomes of the collaborative activities between biomedical industries and research centres

The feasibility of an indicator refers to the possibility and burden of measuring it. It includes conditions such as data availability, comparability of data across cancer centres and countries, and burden of data collection. The validity of an indicator means its capacity to measure what it is intended to measure.

Indicator: Number of public-private partnerships

Definition : Number of public-private partnership. A public-private partnership is a long term agreement between an academic research institute and an industrial partner

124. Do you understand this indicator?

○ Yes

○ No

125. Feasibility

1 (definitely not feasible)	2	3	4	5	6	7	8	9 (definitely feasible)
○	○	○	○	○	○	○	○	○

126. Validity

1 (definitely not valid)	2	3	4	5	6	7	8	9 (definitely valid)
○	○	○	○	○	○	○	○	○

127. Comments

Indicator: Number of papers co-authored with the industry

Definition : Number of articles published by an institution over a period of 3 years that include at least one author affiliated to a health industry

128. Do you understand this indicator?

- Yes
- No

129. Feasibility

1 (definitely not feasible)	2	3	4	5	6	7	8	9 (definitely feasible)
○	○	○	○	○	○	○	○	○

130. Validity

1 (definitely not valid)	2	3	4	5	6	7	8	9 (definitely valid)
○	○	○	○	○	○	○	○	○

131. Comments

Indicator: Number of patents

Definition : Number of patents applied for or approved over a period of 3 years

132. Do you understand this indicator?

- Yes
- No

133. Feasibility

1 (definitely not feasible)	2	3	4	5	6	7	8	9 (definitely feasible)
○	○	○	○	○	○	○	○	○

134. Validity

1 (definitely not valid)	2	3	4	5	6	7	8	9 (definitely valid)
○	○	○	○	○	○	○	○	○

135. Comments

Indicator: Number of patent citations

Definition : Number of patents from an institutions that are cited in subsequent patent applications in the last 3 years

136. Do you understand this indicator?

- Yes
- No

137. Feasibility

1 (definitely not feasible)	2	3	4	5	6	7	8	9 (definitely feasible)
○	○	○	○	○	○	○	○	○

138. Validity

1 (definitely not valid)	2	3	4	5	6	7	8	9 (definitely valid)
○	○	○	○	○	○	○	○	○

139. Comments

Indicator: Patent h-index

Definition : Indicator that combines the number of patents and the patent citation count over a period of 3 years

140. Do you understand this indicator?

- Yes
- No

141. Feasibility

1 (definitely not feasible)	2	3	4	5	6	7	8	9 (definitely feasible)
○	○	○	○	○	○	○	○	○

142. Validity

1 (definitely not valid)	2	3	4	5	6	7	8	9 (definitely valid)
○	○	○	○	○	○	○	○	○

Indicators of research activity, output and outcomes

143. Comments

Indicator: Citation of research in patents

Definition : Number of patents applied for in the last 3 years that cite research produced by an institution

144. Do you understand this indicator?

- Yes
- No

145. Feasibility

1 (definitely not feasible)	2	3	4	5	6	7	8	9 (definitely feasible)

146. Validity

1 (definitely not valid)	2	3	4	5	6	7	8	9 (definitely valid)

147. Comments

Indicator selection

*148. Please select and rank the most important indicators in that category (max. 5)

	1 (Most important indicator)	2	3	4	5 (Least important indicator)
Number of public-private partnerships					
Number of papers co-authored with the industry					
Number of patents					
Number of patent citations					
Patent h-index					
Citation of research in patents					

Bibliometric indicators

Definition: Bibliometric indicators measure the production of a research institute based on their publication record

Indicators of research activity, output and outcomes

The feasibility of an indicator refers to the possibility and burden of measuring it. It includes conditions such as data availability, comparability of data across cancer centres and countries, and burden of data collection. The validity of an indicator means its capacity to measure what it is intended to measure.

Indicator: Number of publications

Definition : Number of peer-reviewed publications authored by the institution over a period of 3 years

149. Do you understand this indicator?

- Yes
- No

150. Feasibility

1 (definitely not feasible)	2	3	4	5	6	7	8	9 (definitely feasible)
○	○	○	○	○	○	○	○	○

151. Validity

1 (definitely not valid)	2	3	4	5	6	7	8	9 (definitely valid)
○	○	○	○	○	○	○	○	○

152. Comments

Indicator: Number of citations

Definition : Number of citations received by a group of researchers from published articles over the last 3 years

153. Do you understand this indicator?

- Yes
- No

154. Feasibility

1 (definitely not feasible)	2	3	4	5	6	7	8	9 (definitely feasible)
○	○	○	○	○	○	○	○	○

155. Validity

1 (definitely not valid)	2	3	4	5	6	7	8	9 (definitely valid)
○	○	○	○	○	○	○	○	○

156. Comments

Indicator: Mean citations per article

Definition : The mean number of citation per articles published in the last 3 years

157. Do you understand this indicator?

- Yes
- No

158. Feasibility

1 (definitely not feasible)	2	3	4	5	6	7	8	9 (definitely feasible)
○	○	○	○	○	○	○	○	○

159. Validity

1 (definitely not valid)	2	3	4	5	6	7	8	9 (definitely valid)
○	○	○	○	○	○	○	○	○

160. Comments

Indicator: H-index for institutions

Definition : The h-index combines the number of articles produced by a research units and the number of its citations. We propose to calculate it over a period of 3 years

161. Do you understand this indicator?

- Yes
- No

162. Feasibility

1 (definitely not feasible)	2	3	4	5	6	7	8	9 (definitely feasible)
○	○	○	○	○	○	○	○	○

163. Validity

1 (definitely not valid)	2	3	4	5	6	7	8	9 (definitely valid)
○	○	○	○	○	○	○	○	○

164. Comments

Indicator: Journal impact factor

Definition : The journal impact factor is the ratio of the number of citations to number of citable items of a journal over a period of 2 years. We propose to calculate the mean impact factor of publications by an institutions over a period of 3 years.

165. Do you understand this indicator?

- Yes
- No

166. Feasibility

1 (definitely not feasible)	2	3	4	5	6	7	8	9 (definitely feasible)
◯	◯	◯	◯	◯	◯	◯	◯	◯

167. Validity

1 (definitely not valid)	2	3	4	5	6	7	8	9 (definitely valid)
◯	◯	◯	◯	◯	◯	◯	◯	◯

168. Comments

Indicator: z-factor

Definition : The z-index takes into account both the number of publications and the impact factor of the journals in which they are published. We propose to calculate it over a period of 3 years

169. Do you understand this indicator?

- Yes
- No

170. Feasibility

1 (definitely not feasible)	2	3	4	5	6	7	8	9 (definitely feasible)
◯	◯	◯	◯	◯	◯	◯	◯	◯

Indicators of research activity, output and outcomes

171. Validity

1 (definitely not valid)	2	3	4	5	6	7	8	9 (definitely valid)
○	○	○	○	○	○	○	○	○

172. Comments

Indicator: Number of publications in top-ranked journals

Definition : Number of publications in highest quality journals of the discipline according to their impact factor. We propose to calculate it over a period of 3 years

173. Do you understand this indicator?

○ Yes

○ No

174. Feasibility

1 (definitely not feasible)	2	3	4	5	6	7	8	9 (definitely feasible)
○	○	○	○	○	○	○	○	○

175. Validity

1 (definitely not valid)	2	3	4	5	6	7	8	9 (definitely valid)
○	○	○	○	○	○	○	○	○

176. Comments

Indicator: Number of highly cited publications

Definition : Number of articles produced by an institution that have a citation count above a certain threshold over a period of 3 years.

177. Do you understand this indicator?

○ Yes

○ No

178. Feasibility

1 (definitely not feasible)	2	3	4	5	6	7	8	9 (definitely feasible)
○	○	○	○	○	○	○	○	○

179. Validity

1 (definitely not valid)	2	3	4	5	6	7	8	9 (definitely valid)
◯	◯	◯	◯	◯	◯	◯	◯	◯

180. Comments

Indicator: Crown indicator

Definition : Average number of received citations divided by the average number that could be expected for publication of the same type published on journals of the same type. We propose to calculate it over a period of 3 years

181. Do you understand this indicator?

◯ Yes

◯ No

182. Feasibility

1 (definitely not feasible)	2	3	4	5	6	7	8	9 (definitely feasible)
◯	◯	◯	◯	◯	◯	◯	◯	◯

183. Validity

1 (definitely not valid)	2	3	4	5	6	7	8	9 (definitely valid)
◯	◯	◯	◯	◯	◯	◯	◯	◯

184. Comments

Indicator: SP-index

Definition : Index that incorporates number of papers, number of citations and impact factor of publications. We propose to calculate it over a period of 3 years

185. Do you understand this indicator?

◯ Yes

◯ No

186. Feasibility

1 (definitely not feasible)	2	3	4	5	6	7	8	9 (definitely feasible)
◯	◯	◯	◯	◯	◯	◯	◯	◯

187. Validity

1 (definitely not valid)	2	3	4	5	6	7	8	9 (definitely valid)
○	○	○	○	○	○	○	○	○

188. Comments

Indicator: b-index

Definition : Number of papers of a scientist that belong to the top 10% of papers in that field. We propose to calculate it over a period of 3 years

189. Do you understand this indicator?

- ○ Yes
- ○ No

190. Feasibility

1 (definitely not feasible)	2	3	4	5	6	7	8	9 (definitely feasible)
○	○	○	○	○	○	○	○	○

191. Validity

1 (definitely not valid)	2	3	4	5	6	7	8	9 (definitely valid)
○	○	○	○	○	○	○	○	○

192. Comments

Indicator: Mean citations per papers

Definition : The mean of citation per articles in the last 3 years.

193. Do you understand this indicator?

- ○ Yes
- ○ No

194. Feasibility

1 (definitely not feasible)	2	3	4	5	6	7	8	9 (definitely feasible)
○	○	○	○	○	○	○	○	○

Indicators of research activity, output and outcomes

195. Validity

definitely not valid								definitely valid
○	○	○	○	○	○	○	○	○

196. Comments

Indicator: j-index

Definition : The j-index is derived from the h-index but also takes into account excess of publications in the h-core and the distribution of citations. We propose to calculate it over a period of 3 years

197. Do you understand this indicator?

- ○ Yes
- ○ No

198. Feasibility

1 (definitely not feasible)	2	3	4	5	6	7	8	9 (definitely feasible)
○	○	○	○	○	○	○	○	○

199. Validity

1 (definitely not valid)	2	3	4	5	6	7	8	9 (definitely valid)
○	○	○	○	○	○	○	○	○

200. Comments

Indicator: e-index

Definition : An h-index with ignored excess citations. We propose to calculate it over a period of 3 years

201. Do you understand this indicator?

- ○ Yes
- ○ No

202. Feasibility

1 (definitely not feasible)	2	3	4	5	6	7	8	9 (definitely feasible)
○	○	○	○	○	○	○	○	○

Indicators of research activity, output and outcomes

203. Validity

1 (definitely not valid)	2	3	4	5	6	7	8	9 (definitely valid)
○	○	○	○	○	○	○	○	○

204. Comments

Indicator: w-index

Definition : If all the papers of a scientist are ranked in descending order of the number of citations they received, the w-index is the highest number of papers one has that have each received at least 10w or more citations

205. Do you understand this indicator?

○ Yes

○ No

206. Feasibility

1 (definitely not feasible)	2	3	4	5	6	7	8	9 (definitely feasible)
○	○	○	○	○	○	○	○	○

207. Validity

1 (definitely not valid)	2	3	4	5	6	7	8	9 (definitely valid)
○	○	○	○	○	○	○	○	○

208. Comments

Indicator: Central index

Definition : Indicator that corrects biases of mass collaboration and punctual success by not considering all production and impact

209. Do you understand this indicator?

○ Yes

○ No

210. Feasibility

1 (definitely not feasible)	2	3	4	5	6	7	8	9 (definitely feasible)
○	○	○	○	○	○	○	○	○

211. Validity

1 (definitely not valid)	2	3	4	5	6	7	8	9 (definitely valid)
○	○	○	○	○	○	○	○	○

212. Comments

Indicator: x-index

Definition : Index calculated from the number of national articles in top 1% and 0.1% of highly cited articles over a period of 3 years.

213. Do you understand this indicator?

○ Yes

○ No

214. Feasibility

1 (definitely not feasible)	2	3	4	5	6	7	8	9 (definitely feasible)
○	○	○	○	○	○	○	○	○

215. Validity

1 (definitely not valid)	2	3	4	5	6	7	8	9 (definitely valid)
○	○	○	○	○	○	○	○	○

216. Comments

Indicator: m-index

Definition : The median number of citations received by papers that have a ranking that is equal to or smaller than h

217. Do you understand this indicator?

○ Yes

○ No

218. Feasibility

1 (definitely not feasible)	2	3	4	5	6	7	8	9 (definitely feasible)
○	○	○	○	○	○	○	○	○

219. Validity

1 (definitely not valid)	2	3	4	5	6	7	8	9 (definitely valid)
◯	◯	◯	◯	◯	◯	◯	◯	◯

220. Comments

Indicator: Q^2 index

Definition : The q2 index is the geometric mean of the h-index and the m-index, defined as the square root of the product of the h- and m- indices. We propose to calculate it over a period of 3 years

221. Do you understand this indicator?

◯ Yes

◯ No

222. Feasibility

1 (definitely not feasible)	2	3	4	5	6	7	8	9 (definitely feasible)
◯	◯	◯	◯	◯	◯	◯	◯	◯

223. Validity

1 (definitely not valid)	2	3	4	5	6	7	8	9 (definitely valid)
◯	◯	◯	◯	◯	◯	◯	◯	◯

224. Comments

Indicator: m-quotient

Definition : h-index adjusted for the researcher’s career length. We propose to calculate it over a period of 3 years

225. Do you understand this indicator?

◯ Yes

◯ No

226. Feasibility

1 (definitely not feasible)	2	3	4	5	6	7	8	9 (definitely feasible)
◯	◯	◯	◯	◯	◯	◯	◯	◯

Indicators of research activity, output and outcomes

227. Validity

1 (definitely not valid)	2	3	4	5	6	7	8	9 (definitely valid)
○	○	○	○	○	○	○	○	○

228. Comments

Indicator: AWCR (age-weighted citation ratio)

Definition : The average number of citations for an entire body of work adjusted for the age of each individual paper. We propose to calculate it over a period of 3 years

229. Do you understand this indicator?

○ Yes

○ No

230. Feasibility

1 (definitely not feasible)	2	3	4	5	6	7	8	9 (definitely feasible)
○	○	○	○	○	○	○	○	○

231. Validity

1 (definitely not valid)	2	3	4	5	6	7	8	9 (definitely valid)
○	○	○	○	○	○	○	○	○

232. Comments

Indicator: Mean normalised citation score

Definition : Average number of citations of publications of a university, normalised for differences between scientific fields, between publication years, and document types. We propose to calculate it over a period of 3 years

233. Do you understand this indicator?

○ Yes

○ No

234. Feasibility

1 (definitely not feasible)	2	3	4	5	6	7	8	9 (definitely feasible)
○	○	○	○	○	○	○	○	○

235. Validity

1 (definitely not valid)	2	3	4	5	6	7	8	9 (definitely valid)
○	○	○	○	○	○	○	○	○

236. Comments

Indicator: Citer h-index (Ch-index)

Definition : The number such that for a general group of papers, ch papers are cited by no more than ch different citers

237. Do you understand this indicator?

○ Yes

○ No

238. Feasibility

1 (definitely not feasible)	2	3	4	5	6	7	8	9 (definitely feasible)
○	○	○	○	○	○	○	○	○

239. Validity

1 (definitely not valid)	2	3	4	5	6	7	8	9 (definitely valid)
○	○	○	○	○	○	○	○	○

240. Comments

Indicator: r-index

Definition : h-index revised by weighting four times more the first and last authors. We propose to calculate it over a period of 3 years

241. Do you understand this indicator?

○ Yes

○ No

242. Feasibility

1 (definitely not feasible)	2	3	4	5	6	7	8	9 (definitely feasible)
○	○	○	○	○	○	○	○	○

Indicators of research activity, output and outcomes

243. Validity

1 (definitely not valid)	2	3	4	5	6	7	8	9 (definitely valid)
○	○	○	○	○	○	○	○	○

244. Comments

Indicator: g-index

Definition : A set of papers has a g-index if g is the highest rank such that top g papers have, together, at least g^2 citations. We propose to calculate it over a period of 3 years

245. Do you understand this indicator?

- ○ Yes
- ○ No

246. Feasibility

1 (definitely not feasible)	2	3	4	5	6	7	8	9 (definitely feasible)
○	○	○	○	○	○	○	○	○

247. Validity

1 (definitely not valid)	2	3	4	5	6	7	8	9 (definitely valid)
○	○	○	○	○	○	○	○	○

248. Comments

Indicator: hg-index

Definition : The hg index of a researcher is computed as the geometric mean of his h and g indices. We propose to calculate it over a period of 3 years

249. Do you understand this indicator?

- ○ Yes
- ○ No

250. Feasibility

1 (definitely not feasible)	2	3	4	5	6	7	8	9 (definitely feasible)
○	○	○	○	○	○	○	○	○

Indicators of research activity, output and outcomes

251. Validity

1 (definitely not valid)	2	3	4	5	6	7	8	9 (definitely valid)
○	○	○	○	○	○	○	○	○

252. Comments

Indicator selection

***253. Please select and rank the most important indicators in that category (5 max)**

	1 (Most important indicator)	2	3	4	5 (Least important indicator)
Number of publications	○	○	○	○	○
Number of citations	○	○	○	○	○
Mean citations per article	○	○	○	○	○
H-index for institutions	○	○	○	○	○
Journal impact factor	○	○	○	○	○
z-factor	○	○	○	○	○
Number of publications in top-ranked journals	○	○	○	○	○
Number of highly cited publications	○	○	○	○	○
Crown indicator	○	○	○	○	○
SP-index	○	○	○	○	○
b-index	○	○	○	○	○
Mean citation per papers	○	○	○	○	○
j-index	○	○	○	○	○
e-index	○	○	○	○	○
w-index	○	○	○	○	○
Central index	○	○	○	○	○
x-index	○	○	○	○	○
m-index	○	○	○	○	○
Q^2 index	○	○	○	○	○
m-quotient	○	○	○	○	○
Age-Weighted Citation Ratio	○	○	○	○	○
Mean normalised citation score	○	○	○	○	○
Citer h-index	○	○	○	○	○
r-index	○	○	○	○	○
g-index	○	○	○	○	○
	○	○	○	○	○

g-index	◯	◯	◯	◯	◯
hg-index	◯	◯	◯	◯	◯

Suggestions and comments

254. Suggestions for other indicators measuring research impact (maximum 5)

1

2

3

4

5

255. General comments
