# Supplementary material for: Identifying potential indicators to measure the outcome of translational cancer research: a mixed methods approach
Source: Health Res Policy Syst. 2015 Dec 3;13:72. doi: 10.1186/s12961-015-0060-5 (PMC4669638; doi:10.1186/s12961-015-0060-5)
Supplement: Additional file 3: — Selection of comments from survey participants. (DOC 36 kb) [file 12961_2015_60_MOESM3_ESM.doc]

**Additional file 3: Selection of comments by participants in the first round Delphi survey**

| **Number of clinical trials** |
| --- |
| ‘The phase of the research is relevant to research outcomes affecting validity as an indicator.’  ‘Clinical Trials as well Medical as Radiation Oncology’ |
| **% of patients included in a clinical trial** |
| ‘It could be misleading in some cases: e.g. rare diseases’ |
| **Number of biomarkers identified** |
| ‘It is subjective. I would ask for those that were published in a peer reviewed journal.’  ‘Identified does not mean validated and implemented’ |
| **Number of patients in a clinical trial with biomarker identification** |
| ‘The validity of this indicator would depend on the main objective of the clinical trial. If the trial is for validating biomarkers, yes, it would be valid. If it is for testing a new drug, no’ |
| **Number of biospecimen collected** |
| ‘I would separate the biospecimens that are used for diagnostic and research purposes. This would be a better indicator of research output. Also I would separate blood from tissue (as well as biopsies from tumor mass).’  ‘Less valid than others because it could simply reflect a higher or lower aggressiveness in the diagnostic process, without any real added clinical/research value. Too many invasive procedures are not always sign of higher quality/better care.’ |
| **Number of hypotheses generated** |
| ‘In my opinion, a quite good indicator would be "the number of translational research projects supported by a financing body". I understand that several projects may test the same hypotheses through different approaches, but it will be quite hard to have access to such information within an institution.’ |
| **Citation of research in guidelines** |
| ‘Define in which clinical guidelines: local, regional, national EU, etc only then it might be possible to monitor it’ |
| **Number of citations** |
| ‘3 year is too short.’  ‘Problem for collaborative works related to the fact that the power is not the same if you are in the first authors or not.’ |
| **Institutional h-index** |
| ‘The only problem that I see with this indicator is the period of time : I am not sure that 3 years are suficient to measure a scientific impact’  ‘H-index calculated over a period of only 3 years might a bit limited to see the long-term impact of research’ |
| **Number of publications in top-ranked journals** |
| ‘What is exactly the "journals of the discipline"? (translational research is anyway "biomedical research"... )’  ‘It is important that the top journals at different disciplines have very different impact factors! This needs to be taken into account!’ |
| **Number of database generated** |
| ‘The interest of creating a database for each “translationnal study” is poor (especially with the development of "big data"), it would of interest to measure the capacity of an institution to aggregate all its databases (included clinical and molecular data)’  ‘The number of databases is not valid at all. The tendency should be to centralize information.’ |
